# Associations between antioxidant vitamin intake and mental health in Swedish adolescents: a cross-sectional study

**DOI:** 10.1007/s00394-025-03701-1

**Published:** 2025-05-24

**Authors:** Martina Pensa, Karin Kjellenberg, Emerald Heiland, Örjan Ekblom, Gisela Nyberg, Björg Helgadóttir

**Affiliations:** 1 Department of Physical Activity and Health, The Swedish School of Sport and Health Sciences, Stockholm, Sweden; 2 Department of Surgical Sciences, Medical Epidemiology, Uppsala University, Uppsala, Sweden; 3 Department of Global Public Health, Karolinska Institutet, Solna, Sweden

**Keywords:** Vitamin C, β-carotene, Vitamin E, Anxiety, Psychosomatic, HRQoL

## Abstract

**Purpose:**

Mental health problems are increasingly prevalent during adolescence. Nutritional factors, particularly antioxidants, are of interest due to their potential to reduce oxidative stress and inflammation linked to mental health issues. However, the relationship between dietary antioxidants and adolescent mental health remains unclear. This study aimed to investigate this association in Swedish adolescents and explore potential gender differences.

**Methods:**

Cross-sectional data were gathered among Swedish boys and girls aged 13–14 years (*n* = 1139). Participants reported their dietary intake using a detailed web-based method and mental health outcomes, including anxiety, psychosomatic symptoms, and health-related quality of life (HRQoL) using self-report scales. Multiple linear regression analysis, adjusted for confounders, was used to investigate the associations between mental health outcomes and tertiles of dietary intake of vitamin C, E and β-carotene.

**Results:**

Adolescents in the highest tertile of β-carotene intake reported lower anxiety (β=-1.23, 95% CI=-2.34, -0.12), fewer psychosomatic symptoms (β=-0.91, 95% CI=-1.69, -0.13), and better HRQoL (β = 0.89, 95% CI = 0.11, 1.68). Similarly, higher vitamin C intake was associated with fewer psychosomatic problems (β=-1.00, 95% CI=-1.79, -0.21). Vitamin E intake showed no associations. Significant gender interactions were observed on the multiplicative scale analysis and limited to the middle tertiles of β-carotene and vitamin C for anxiety and psychosomatic symptoms, respectively.

**Conclusion:**

Our findings underscore the potential role of dietary antioxidants, particularly β-carotene and vitamin C, in adolescent mental health. Further research including diverse populations and employing prospective designs could deepen the understanding and inform public health interventions.

**Supplementary Information:**

The online version contains supplementary material available at 10.1007/s00394-025-03701-1.

## Introduction

Mental health conditions remain a global challenge, especially affecting adolescents [1]. Early adolescence is a vulnerable period of life, where the development of mental health conditions is quite common. Approximately 50% of all adult mental disorders begin by age 14, with prevalence of anxiety symptoms emerging before the age of 10 [2, 3]. In Europe, the prevalence of mental disorders among adolescents is over 16%, which is higher than the global average of 13% [4]. Within Europe, studies have shown a more significant rise in mental health problems among Swedish adolescents compared to other Nordic countries [5]. Specifically, self-reported psychosomatic complaints in Sweden have risen dramatically, from 29% in 1985 to 57% in 2013 [6, 7]. In addition, anxiety has been reported as the most prevalent mental health problem among this age group, although data are limited [8].

Psychosomatic complaints are physical symptoms (like pains) that appear to be triggered or worsened by psychological factors (such as mental health complaints) [9]. For example, anxiety manifests through excessive worry, fear, emotional distress (psychological symptoms) but also through headaches and stomach-aches (physical symptoms) [10, 11]. Recent research has shown an association between psychosomatic symptoms and future mental disorders in Swedish adolescents [6]. Notably, girls appear more prone to experiencing these physical expressions of emotional distress compared to boys [7]. These mental health problems can negatively affect adolescents’ health-related quality of life (HRQoL), which is a broad concept that covers a person’s physical, mental, emotional, and social well-being, and their ability to function in daily life [12, 13]. Due to this impact, measures of HRQoL are increasingly used to understand children’s psychosocial functioning, their perceptions of illness, and to identify subgroups of children and adolescents at risk of health problems [13].

Diet quality has been suggested to be a modifiable factor potentially influencing mental health resilience [14]. The available evidence suggests that high-quality dietary patterns rich in fruits, vegetables, whole grains, nuts, seeds, and fish may be linked to lower odds of depression and better HRQoL, also in Swedish adolescents [15–17]. Sex differences in dietary habits may also play a role, with girls generally consuming more fruits and vegetables than boys [18, 19]. Although healthier dietary patterns may offer some protective effects against mental health issues the relationship between diet and mental health is complex and bidirectional. While healthier eating patterns may contribute to better mental health, mental health status can also influence dietary choices, and the exact direction of this association has yet to be firmly established [20].

While research on dietary patterns is important, identifying the role of individual nutrients may offer new insights for future interventions, particularly in younger populations with limited research [21]. A research area called Nutritional Psychiatry tries to identify the important nutrients for mental health and explore their potential for preventing or treating mental health problems [21]. Single nutrients are thought to target and regulate various biological pathways implicated in mental illness, offering potential therapeutic benefits [14, 22]. For instance, dietary antioxidants have garnered interest as important nutrients which fight oxidative stress and inflammation, which are processes also implicated in the development of neuropsychiatric diseases such as anxiety and depression [23–25]. The most familiar dietary antioxidants are vitamin C, E and β-carotene and they are also part of the non-enzymatic antioxidant defence of the biological system [24].

Most studies on antioxidants and mental health have been carried out on adult populations with depressive symptoms, creating a gap for other relevant mental health outcomes and populations [14]. Although evidence remains limited in adolescents, studies indicate an inverse association between antioxidant vitamins like vitamin C and E and depression in different populations [26, 27]. Also, carotenoids seem to reduce the risk of depressive-like symptoms [28]. In addition, a study found that supplementation of vitamin C, E and β-carotene might benefit individuals with clinical anxiety and depression alongside standard medications [24].

These findings highlight the need for research on the association between nutrient intake and diverse mental health outcomes beyond depression and across various populations. This includes non-clinical groups and adolescents, where research is particularly limited. To the best of our knowledge, one previous study explored the association between dietary antioxidant intake (vitamin C, E and β-carotene) and anxiety risk in adolescents [29]. However, the study included only girls, thus limiting its generalizability.

The aim of this study was therefore to investigate the association between the dietary intake of specific antioxidants (vitamin C, E and β-carotene) and mental health outcomes (anxiety symptoms, psychosomatic symptoms and HRQoL) in Swedish adolescents. In addition, we examined gender differences in these associations.

## Methods

### Study design and setting

This study utilised a cross-sectional design. The data were collected from a large study called Physical Activity for Healthy Brain Functions in School Youth, performed in Stockholm, Sweden, between September and December 2019.

### Recruitment and study population

**Fig. 1:**
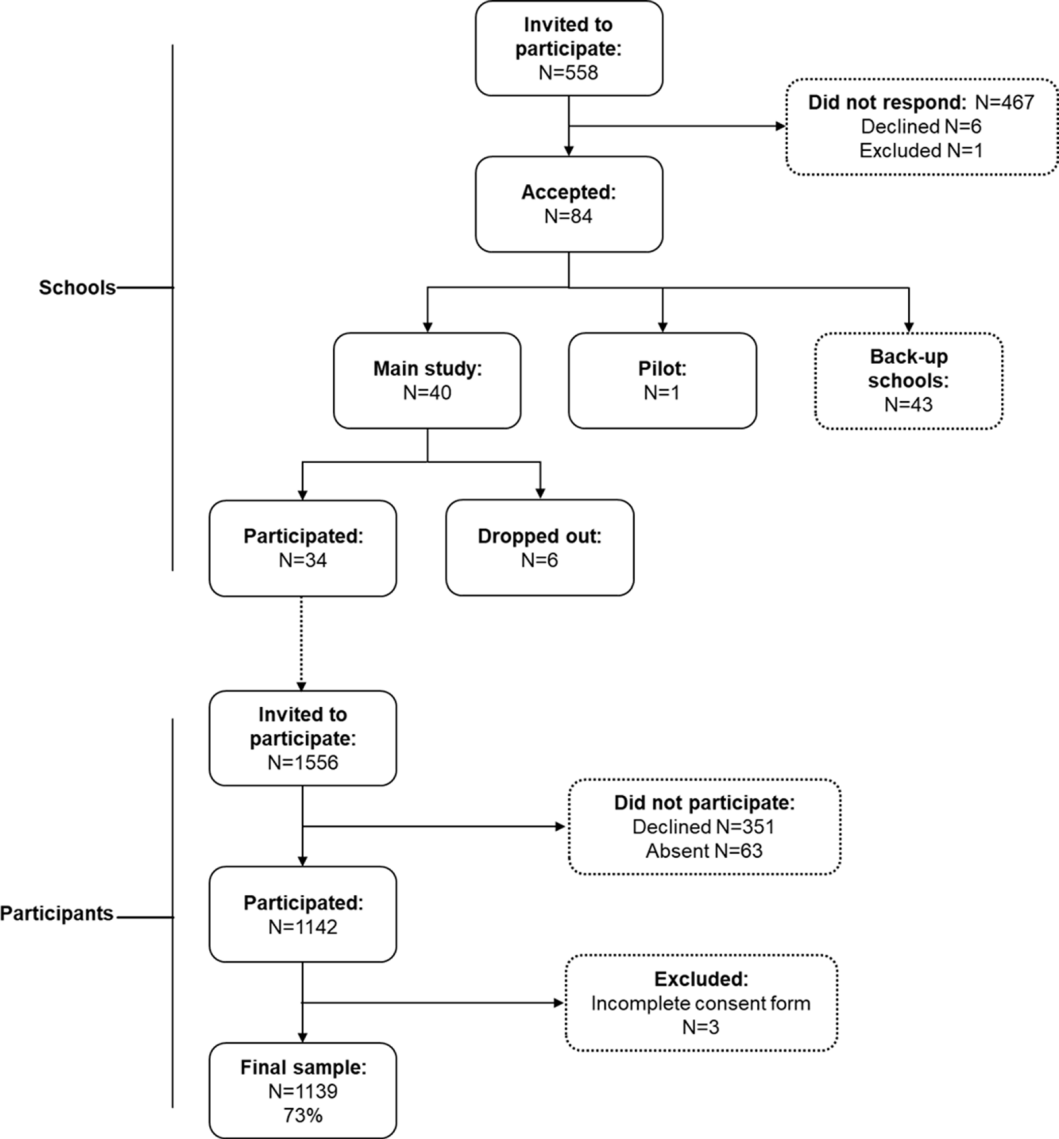
Flowchart showing recruitment of schools and participants

More than 500 schools within a 2–3 h driving radius of the Swedish School of Sport and Health Sciences (GIH) were invited to participate in the study. A total of 34 schools were finally enrolled in the study and the students in the participating classes in grade 7 were invited to take part. The final sample included 1139 students, 49% boys and 51% girls, aged 13–14. Figure 1 gives an overview of the process of recruiting schools and students. A more detailed description of the recruitment process is provided in the study by Nyberg et al. [30]. The students spent half a day at GIH for data collection where data on their dietary intake and mental health were collected. The characteristics of the students are shown in Table 1.

**Table 1:** Descriptive characteristics of the study sample by gender

		All	Girls	Boys	*p*-value
	*n* (missing)	*n* (%) or mean ± SD	*n* (%) or mean ± SD	*n* (%) or mean ± SD	
Number of participants		1139 (100)	580 (51.0)	558 (49.0)	
Age, years	1139 (0)	13.4 ± 0.3	13.4 ± 0.3	13.4 ± 0.4	0.147
Foreign background	1117 (22)				0.534
*Swedish born and at least one Swedish parent*		800 (71.6)	414 (72.5)	386 (70.8)	
*Born outside of Sweden or both parents born outside of Sweden*		317 (28.4)	157 (27.5)	159 (29.2)	
Parental education	1102 (37)				0.674
*≤ 12 years*		372 (33.8)	192 (34.1)	180 (33.5)	
*> 12 years*		730 (66.2)	371 (65.9)	358 (66.5)	
BMI categories	1135 (4)				0.181
*Underweight*		89 (7.8)	38 (6.6)	51 (9.2)	
*Normal weight*		815 (71.8)	430 (74.1)	384 (69.3)	
*Overweight*		179 (15.8)	89 (15.3)	90 (16.2)	
*Obese*		52 (4.6)	23 (4.0)	29 (5.2)	
BMIsds	1134 (5)	0.36 ± 1.23	0.45 ± 1.11	0.26 ± 1.35	0.012
Total energy intake, kcal/d	1133 (6)	2000 ± 847	1844 ± 782	2165 ± 882	< 0.001
Antioxidant vitamin intake	1133 (6)				
*Vitamin C*,* mg*		70 ± 73	68 ± 58	72 ± 85	0.300
*Vitamin E*,* mg*		11 ± 6	10 ± 6	12 ± 7	< 0.001
*β-Carotene*,* mcg*		1528 ± 1953	1473 ± 1883	1579 ± 2018	0.361
Mental health					
*Anxiety (SCAS-S)*	1073 (66)	13.7 ± 7.9	16.6 ± 8.0	10.6 ± 6.6	< 0.001
*Psychosomatic Problems (PSP-scale)*	1096 (43)	9.3 ± 5.5	10.9 ± 5.6	7.7 ± 4.8	< 0.001
*Health-related quality of life (Kidscreen-10)*	1097 (42)	39.6 ± 5.4	38.3 ± 5.2	41.0 ± 5.3	< 0.001

SD, standard deviation; BMI, body mass index; BMIsds, body mass inde × SD score; SCAS-S, Spence Children’s Anxiety Scale; PSP-scale, Psychosomatic Problems Scale

### Dietary recall procedure

To assess dietary intake, students were asked to recall their food consumption over a 3-day period, consisting of two weekdays and one weekend day. The 3-day diet recall consisted of (1) the day before their visit at GIH, (2) day of their visit at GIH (completed the day after their visit), and (3) at home within a week of their visit.

For this study, an average of two days of food records was used for the analysis. The second recall day was excluded because it was the day the students visited the research centre for data collection and their dietary intake might not have reflected their normal dietary habits.

### Dietary assessment method

The participants used a validated web-based tool called RiksmatenFlexDiet to recall their 3-day dietary intake. The method was created by the Swedish Food Agency for the Riksmaten Adolescents 2016/17 study and it employs a repeated 24-hour multiple-pass recall protocol [31]. The list of food was obtained from the Swedish Food Agency database (Riksmaten Adolescents 2016/17 version) which allowed for the calculation of estimated macro- and micronutrient amounts based on the reported dietary data.

**Fig. 2:**
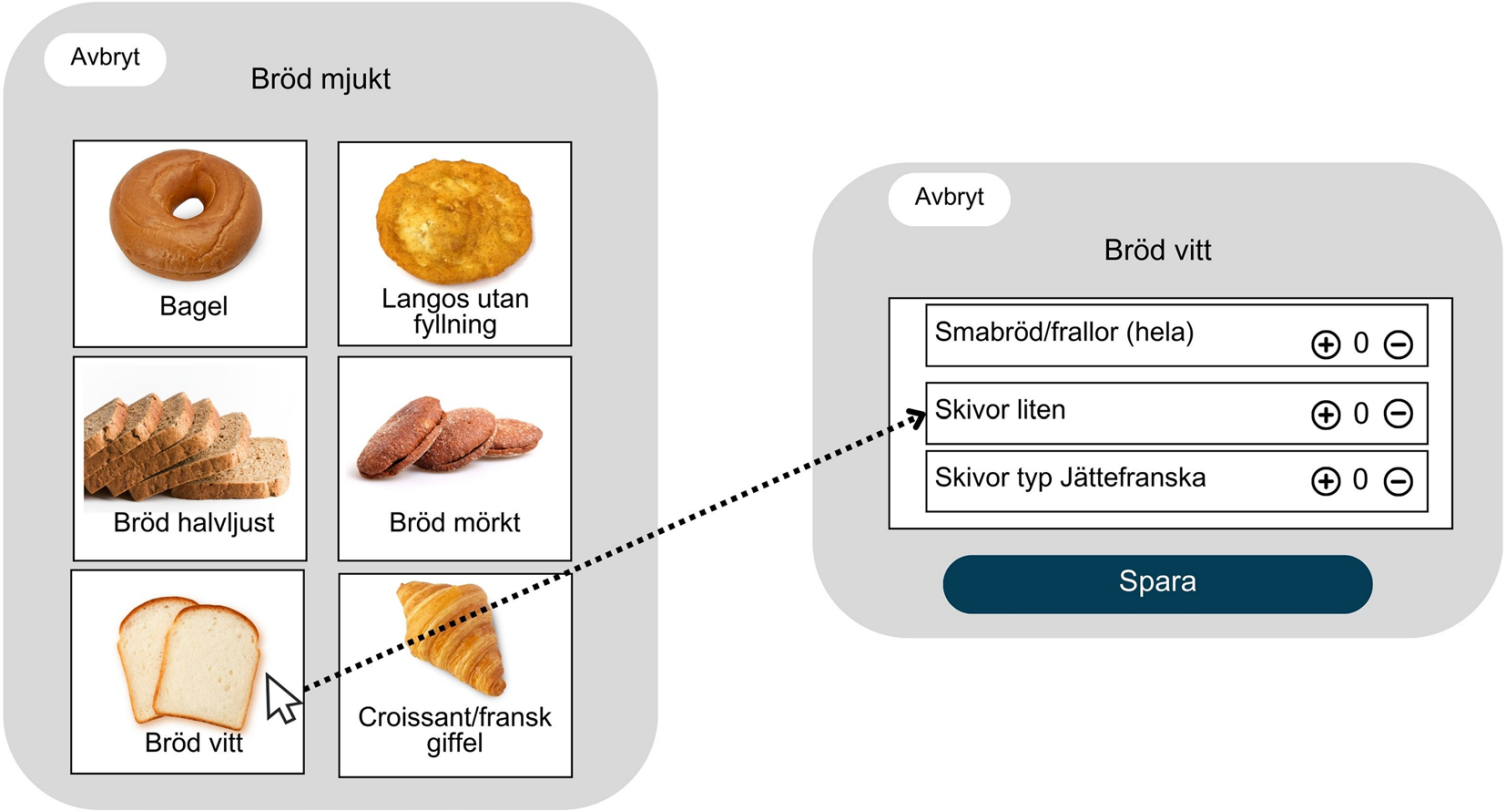
Visual representation of some of the bread options and portions in RiksmatenFlexDiet. The left panel shows the main categories of soft bread, including bagels, langos (a Hungarian fried bread), medium-light bread, dark bread, white bread, and croissants/French rolls. The right panel shows subcategories of white bread, allowing users to choose between small rolls, small slices, or large slices (“Jättefranska”). Users can add or remove items using the “+” and “−” buttons and save their selection by pressing “Spara” (Save)

Notably, RiksmatenFlexDiet allows either single ingredients selection, such as an apple, or complete meals, such as lasagna and it provides a visual portion size guide for each of the 39 food categories. It also includes pictures of common foods to help participants choose and register their intake accurately. Figure 2 shows some of the available options and portions for bread in RiksmatenFlexDiet. Finally, participants had the opportunity to review all their entries before completing the registration.

### Anxiety symptoms assessment

Anxiety symptoms were self-reported by the students using a short version of the Spence Children’s Anxiety Scale (SCAS-S). It contains 19 items that cover five subscales aimed at assessing different types of anxiety disorders (for example: “I worry that something bad will happen to me”) [32]. The responses to the items are scored on a 4-point Likert scale ranging from “never” to “always” [32]. The final score is calculated by summing the single scores from each item, and it ranges from 0 to 57; higher values reflect more anxiety symptoms [32].

### Psychosomatic problems assessment

The students’ psychosomatic health was measured using a self-report scale called Psychosomatic Problems (PSP)-scale. It consists of eight questions about psychosomatic symptoms experienced in the last year, such as “have you felt tense?” [33]. The response categories are ranging from “never” to “always” [33]. These response options are coded from 0 to 4 [33]. The final score is the result of the summation of the score for each item, with a minimum score of 0 and a maximum score of 32; higher scores reflect more psychosomatic problems [33].

### Assessment of health-related quality of life (HRQoL)

The participants’ general HRQoL was self-reported using a short scale called Kidscreen-10. It measures the subjective health perception of children and adolescents aged 8–18 years old and consists of 10 Likert scale questions which provide a global score of HRQoL [12]. The questions refer to the previous week, such as “have you got on well at school?” [12]. The answer options for the 10 questions range from “not at all” to “extremely” on a 5-point Likert scale and are coded from 1 to 5 [12]. The scores for each item were added up according to the Kidscreen manual. The result is a scale from 10 to 50 where high scores are indicative of a better HRQoL [12].

### Other variables

Participants provided information about their gender (“boy,” “girl,” or “other”) and country of birth (“Swedish born with at least one Swedish parent” or “Born outside Sweden or both parents born outside Sweden”). Since only one student chose “other” for gender, this observation was not included in the stratified analysis. Parental education data were obtained from register data provided by Statistics Sweden, and it was grouped into ≤ 12 years and > 12 years, using the parent with the highest education. Anthropometric measurements (height and weight) were collected, and BMI (body mass index) categories were calculated based on international guidelines [34]. Furthermore, BMI standard deviation score (BMIsds) was calculated. This estimate results by comparing a child’s BMI to the average BMI for Swedish children of the same age and sex [35]. Finally, the total energy intake of the participants was calculated using the food records as previously explained. Total energy intake, parental education, BMIsds, and country of birth were considered as potential confounders, while gender as potential effect modifier, based on a Directed Acyclic Graph (Supplementary Material 1).

### Statistical methods

All data processing and the statistical analyses were performed using IBM SPSS Statistics 28.0. Continuous variables are presented using means with standard deviations (SD), while categorical variables are described in counts and percentages. Comparisons of the characteristics between girls and boys were performed using independent t-tests for continuous variables and χ2 for categorical variables. Participants were categorized based on tertiles of vitamin C, E and β-carotene intakes. The cross-sectional relationship between the tertiles of antioxidant intakes and the continuous outcome variables (SCAS-S, PSP-scale, and Kidscreen-10) was tested with multiple linear regression models. The first tertile was considered as the reference category. Continuous dependent variables were checked for normality through histogram and boxplot charts, skewness, kurtosis and Kolmogorov-Smirnov analysis. Despite a skewed distribution of antioxidant data, the assumptions for the regression analysis were met due to normally distributed residuals. Model 1 represented the crude model, while Model 2 included adjustments for gender, BMIsds, and total energy intake. Although various confounders were initially considered for their potential effect on both vitamin intake and mental health outcomes, only three variables were retained in the final model as they significantly improved model fit. The analysis was then stratified by gender as mental health outcomes were significantly different between girls and boys. To ensure equal distribution within each gender group for the stratified analysis, tertiles for antioxidant intake were recalculated separately for girls and boys. The results from crude models are provided for reference, consequently the discussion will be focused on the adjusted models for more accurate association estimates. In addition, an adjusted interaction analysis on the multiplicative scale was performed and on the additive scale using the PROCESS macro 4.2 in SPSS [36]. Finally, a summative score was created based on adherence to the Average Requirements (AR) for vitamin C, vitamin E, and β-carotene stated in the Nordic Nutrition Recommendations 2023 for 13-year-olds. Participants received a score of 0 if none of the recommended intakes were met, 1 if they met one, 2 if they met two, and 3 if they met al.l three. The score of 0 was used as the reference category in the regression analyses, which examined the association of the score with mental health outcomes, adjusting for gender, BMIsds, and total energy intake. Assumptions for regression were checked for all regression analyses, including normality, linearity, homoscedasticity, and absence of multicollinearity. The level of statistical significance was set at *p* < 0.05.

## Results

### Sample characteristics

The characteristics of the participants are presented in Table 1. The final sample included 1139 students, evenly distributed between girls (51%) and boys (49%), with a mean age of 13.4 ± 0.3 years. Over 70% of the participants were Swedish born and with at least one Swedish born parent and over 66% had at least one parent with advanced education (≥ 12 years). In terms of BMI, most of the students (71.8%) fell within the normal weight group, with approximately 15% categorised as overweight.

A total of 1036 out of 1139 participants completed 2-days of food record (i.e. the first and the third recall day) and were included in the statistical analyses. For 86% of the students, the first recall day was a weekday, and around half of the students registered the third recall day being a weekend day. The average antioxidant intake, except for vitamin E, showed no significant difference between girls and boys (Table 1). Boys had a slightly higher vitamin E intake (12 ± 7 mg/day) compared to girls (10 ± 6 mg/day). However, boys had also higher caloric intake compared to girls (2165 ± 882 vs. 1844 ± 782 kcal/day). According to the recommended AR for this age group, on average, girls did not reach the AR for vitamin C (75 mg/day) and β-carotene (3,060 mcg/day), while boys met the AR for vitamin C (65 mg/day) but did not reach the AR for β-carotene (3,060 mcg/day). Both met the recommendations for vitamin E (8 mg/day for girls, 9 mg/day for boys) [37].

A total of 1039 out of 1139 students completed all three self-reported mental health assessments. Table 1 shows that girls had significantly higher scores for the two negative mental health outcomes, compared to boys, with 17 ± 8 for anxiety and 11 ± 6 for psychosomatic problems. Moreover, girls had a lower HRQoL score (38 ± 5), compared to boys who had 41 ± 5.

### Associations between antioxidant intake and mental health outcomes in the whole sample

The associations between antioxidant intake (vitamin C, vitamin E and β-carotene) and anxiety symptoms are shown in Table 2. The analysis adjusted for gender, BMIsds and total energy intake revealed significant results for the students with the highest intake of β-carotene, compared to those in the lowest tertile. The students with the highest β-carotene intake reported 1.23 units lower (95% CI:-2.34, -0.12) anxiety scores, compared to those in the lowest tertile group. On the other hand, there were no significant associations between vitamin E and C intake and anxiety symptoms.

**Table 2:** Linear regression models for the association between tertiles of antioxidant intakes and anxiety scores (SCAS-S)

	All			Girls			Boys		
	Tertiles of vitamin intake			Tertiles of vitamin intake			Tertiles of vitamin intake		
	T1	T2	T3	T1	T2	T3	T1	T2	T3
		β (95% CI)			β (95% CI)			β (95% CI)	
Vitamin C									
*Model 1* ^*a*^	Ref	-0.59 (-1.66,0.51)	-0.36 (-1.46,0.77)	Ref	-1.13 (-2.77,0.51)	-0.30 (-1.93,1.34)	Ref	-0.66 (-2.05,0.73)	-0.84 (-2.23,0.54)
*Model 2* ^*b*^	Ref	-0.57 (-1.65,0.52)	-0.35 (-1.41,0.82)	Ref	-1.04 (-2.69,0.61)	-0.08 (-1.77,1.62)	Ref	-0.50 (-1.91,0.91)	-0.55 (-1.98,0.89)
Vitamin E									
*Model 1* ^*a*^	Ref	-0.66 (-1.81,0.50)	-1.99* **(-3.15**,**-0.83)**	Ref	-0.40 (-2.03,1.24)	-0.49 (-2.13,1.15)	Ref	-1.63* **(-3.01**,**-0.25)**	-1.48* **(-2.86**,**-0.11)**
*Model 2* ^*b*^	Ref	-0.32 (-1.46,0.83)	-0.81 (-2.24,0.63)	Ref	-0.13 (-1.88,1.62)	0.27 (-1.85,2.39)	Ref	-1.48 (-2.97,0.01)	-1.25 (-3.10,0.61)
β-Carotene									
*Model 1* ^*a*^	Ref	-0.93 (-2.09,0.23)	-1.37* **(-2.53**,**-0.21)**	Ref	-1.04 (-2.68,0.60)	-1.34 (-2.97,0.30)	Ref	-1.16 (-2.54,0.22)	-1.60* **(-2.98**,**-0.21)**
*Model 2* ^*b*^	Ref	-0.66 (-1.75,0.44)	-1.23* **(-2.34**,**-0.12)**	Ref	-0.96 (-2.63,0.70)	-1.17 (-2.85,0.51)	Ref	-1.00 (-2.42,0.41)	-1.34 (-2.79,0.11)

Ref, reference; CI, confidence interval

^a^ Crude model. All: *n* = 1073; Girls: *n* = 547; Boys: *n* = 525

^b^ Adjusted for gender, BMIsds and total energy intake. All: *n* = 1068; Girls: *n* = 547; Boys: *n* = 521

* Significant results (*p* < 0.05)

Table 3 shows the associations between antioxidant intake (vitamin C, vitamin E and β-carotene) and psychosomatic problems. The findings revealed significant results for the participants in the middle and highest intake of vitamin C and β-carotene, compared to those with the lowest intake. The adjusted association for the highest intake of vitamin C translated to a lower score of 1 unit in the PSP-scale (95% CI:-1.79, -0.21), compared to the lowest tertile. The adjusted association between the group in the highest intake of β-carotene and psychosomatic problems translated to a beta coefficient of -0.91 (95% CI:-1.69, -0.13). There were no significant associations between vitamin E and psychosomatic symptoms after adjusting for gender, BMIsds, and total energy intake.

**Table 3:** Linear regression models for the association between tertiles of antioxidant intakes and scores of psychosomatic problems (PSP-scale)

	All			Girls			Boys		
	Tertiles of vitamin intake			Tertiles of vitamin intake			Tertiles of vitamin intake		
	T1	T2	T3	T1	T2	T3	T1	T2	T3
		β (95% CI)			β (95% CI)			β (95% CI)	
Vitamin C									
*Model 1* ^*a*^	Ref	-0.96* **(-1.76**,**0.17)**	-1.22* **(-2**,**01-0.42)**	Ref	-0.67 (-1.80,0.46)	-1.01 (-2.14,0.13)	Ref	-1.00 (-2.01,0.00)	-1.63* **(-2.64**,**-0.62)**
*Model 2* ^*b*^	Ref	-0.85* **(-1.61**,**0.09)**	-1.00* **(-1.79**,**-0.21)**	Ref	-0.47 (-1.61,0.66)	-0.49 (-1.66,0.68)	Ref	-0.88 (-1.90,0.13)	-1.44* **(-2.48**,**0.39)**
Vitamin E									
*Model 1* ^*a*^	Ref	-0.94* **(-1.72**,**-0.15)**	-1.71* **(-2.50**,**-0.91)**	Ref	-2.00* **(-3.11**,**-0.88)**	-1.20* **(-2.32**,**-0.08)**	Ref	-0.71 (-1.72,0.30)	-1.05* **(-2.06**,**-0.50)**
*Model 2* ^*b*^	Ref	-0.47 (-1.28,0.33)	-0.55 (-1.56,0.47)	Ref	-1.39* **(-2.59**,**-0.20)**	0.14 (-1.31,1.58)	Ref	-0.50 (-1.59,0.59)	-0.83 (-2.19,0.53)
β-Carotene									
*Model 1* ^*a*^	Ref	-1.17* **(-1.96**,**-0.38)**	-1.21* **(-2.00**,**-0.41)**	Ref	-1.41* **(-2.54**,**-0.29)**	-1.53* **(-2.65**,**-0.40)**	Ref	-1.06* **(-2.06**,**-0.06)**	-0.91 (-1.92,0.10)
*Model 2* ^*b*^	Ref	-0.88* **(-1.64**,**-0.11)**	-0.91* **(-1.69**,**-0.13)**	Ref	-1.14* **(-2.28**,**-0.01)**	-1.15 (-2.30,0.00)	Ref	-0.91 (-1.93,0.12)	-0.61 (-1.67,0.44)

Ref, reference; CI, confidence interval

^a^ Crude model. All: *n* = 1096; Girls: *n* = 563; Boys: *n* = 532

^b^ Adjusted for gender, BMIsds and total energy intake. All: *n* = 1091; Girls: *n* = 563; Boys: *n* = 528

* Significant results (*p* < 0.05)

Table 4 shows the results from the linear regression between antioxidant intake (vitamin C, vitamin E and β-carotene) and HRQoL. Significant associations were observed among students with higher β-carotene intake, compared to those with the lowest intake. This association translated to a higher score for HRQoL of 1.02 units (95% CI:0.25, 1.80) for the students in the middle tertile and of 0.89 (95% CI:0.11, 1.68) for the ones in the highest intake group. In contrast, vitamin E and C intake did not show significant associations with the outcome.

**Table 4:** Linear regression models for the association between tertiles of antioxidant intakes and HRQoL scores (Kidscreen-10)

	All			Girls			Boys		
	Tertiles of vitamin intake			Tertiles of vitamin intake			Tertiles of vitamin intake		
	T1	T2	T3	T1	T2	T3	T1	T2	T3
		β (95% CI)			β (95% CI)			β (95% CI)	
Vitamin C									
*Model 1* ^*a*^	Ref	0.57 (-0.22,1.36)	0.66 (-0.13,1.45)	Ref	0.90 (-0.16,1.96)	0.77 (-0.30,1.83)	Ref	0.29 (-0.81,1.40)	0.59 (-0.53,1.70)
*Model 2* ^*b*^	Ref	0.49 (-0.28,1.29)	0.54 (-0.26,1.33)	Ref	0.81 (-0.26,1.87)	0.54 (0.56,1.64)	Ref	0.18 (-0.95,1.29)	0.38 (-0.77,1.54)
Vitamin E									
*Model 1* ^*a*^	Ref	0.48 (-0.30,1.27)	0.93* **(0.15**,**1.72)**	Ref	0.49 (-0.57,1.54)	0.24 (-0.83,1.30)	Ref	1.29* (0.20,**2.39)**	0.24 (-0.86,1.34)
*Model 2* ^*b*^	Ref	0.16 (-0.66,0.97)	0.20 (-0.83,1.22)	Ref	0.08 (-1.06,1.22)	-0.46 (-1.85,0.93)	Ref	0.93 (-0.26, 2.11)	-0.37 (-1.84,1.10)
β-Carotene									
*Model 1* ^*a*^	Ref	1.20* (0.42,**1.99)**	1.05* **(0.26**,**1.83)**	Ref	1.13* **(0.08**,**2.19)**	1.55* (0.50,**2.60)**	Ref	1.12* (0.02,**2.22)**	0.46 (-0.65,1.56)
*Model 2* ^*b*^	Ref	1.02* **(0.25**,**1.80)**	0.89* **(0.11**,**1.68)**	Ref	1.02 (-0.50,2.09)	1.46* (0.38,**2.54)**	Ref	1.01 (-0.11,2.14)	0.21 (-0.95,1.36)

Ref, reference; CI, confidence interval

^a^ Crude model. All: *n* = 1097; Girls: *n* = 562; Boys: *n* = 534

^b^ Model 2: adjusted for gender, BMIsds and total energy intake. All: *n* = 1093; Girls: *n* = 562; Boys: *n* = 531

* Significant results (*p* < 0.05)

Supplementary scatter plots (Supplementary Material 2) provide a visual representation of the distribution of mental health scores across tertiles of vitamin C, vitamin E, and β-carotene intake in the whole sample.

### Gender stratified analysis

Girls in the middle tertile of vitamin E and β-carotene intake showed significant associations with psychosomatic problems in the model adjusted for BMIsds and total energy intake. The girls had a lower PSP-scale score compared to those in the lowest tertile, which translated to a beta coefficient of -1.39 (95% CI:-2.59, -0.20) for vitamin E and of -1.14 (95% CI:-2.28, -0.01) for β-carotene. In contrast, boys showed a significant association with vitamin C intake in the highest tertile (adjusted β coefficient:-1.44 [95% CI:-2.48, 0.39]). Girls in the highest tertile of β-carotene intake had also a better HRQoL score (adjusted β:1.46 [95% CI:0.38, 2.54]).

To assess whether gender moderated the associations, multiplicative and additive interaction analyses were conducted with adjustments for confounders. Significant multiplicative interactions with gender were observed in the middle tertile of β-carotene and vitamin C intake, for anxiety (β:-0.37 95% CI: -0.73, -0.02) and psychosomatic symptoms (β:-0.31, 95% CI:-0.56, -0.06), respectively. However, the interaction analysis on the additive scale did not show significant results. Detailed results of the adjusted interaction analyses are provided in the Supplementary Material 3.

### Associations between vitamin intake score and mental health outcomes

Table 1 in the Supplementary Material 4 shows the linear regression analysis of the summative vitamin intake score and mental health outcomes (anxiety, psychosomatic symptoms, HRQoL) adjusted for gender, BMIsds and total energy intake. A higher vitamin intake score was significantly associated with fewer psychosomatic symptoms in the full sample and among boys. In the full sample, participants in Score 1 (β:-0.91, 95% CI:-1.72, -0.09), Score 2 (β:-1.19, 95% CI:-2.16, -0.23), and Score 3 (β:-1.73, 95% CI:-3.16, -0.29) reported fewer psychosomatic symptoms compared to those in the reference group (Score 0). Similarly, among boys, significant negative associations were observed for those in Score 1 (β:-1.15, 95% CI:-2.24, -0.07), Score 2 (β:-1.76, 95% CI:-3.03, -0.49), and Score 3 (β:-2.48, 95% CI:-4.29, -0.66) compared to the students not meeting any of the recommended vitamin intakes. No significant associations were found for anxiety, HRQoL in the whole sample or among boys, and no associations were seen for girls for any of the outcomes.

## Discussion

The aim of the study was to investigate the cross-sectional associations between antioxidants (vitamin C, E, and β-carotene) and mental health outcomes (anxiety, psychosomatic problems and HRQoL) in a sample of 1139 Swedish adolescents. We found that higher β-carotene intake was associated with all three mental health outcomes after adjusting for confounders: lower anxiety scores, fewer psychosomatic symptoms, and higher HRQoL scores. On the other hand, a higher intake of vitamin C showed an association with fewer psychosomatic problems. In contrast, vitamin E showed no significant associations with the outcomes in the adjusted models. Finally, gender interactions were predominantly non-significant, except for significant multiplicative interactions observed in the middle tertiles of β-carotene (for anxiety) and vitamin C (for psychosomatic symptoms).

The present findings on β-carotene align with the limited research available. One study on antioxidant intake and anxiety in adolescents, conducted by Farhadnejad et al. (2020), found that higher β-carotene intake was linked with a lower risk of depression, anxiety, and stress in Iranian girls [29]. Similar to the present study, no significant associations with vitamin E and vitamin C intake and anxiety were observed. Considering that the present study design included both girls and boys, this allows for a broader perspective on the associations between β-carotene and mental health, compared to the study by Farhadnejad et al. (2020) which investigated only girls.

The observed association between β-carotene and mental health outcomes underscores the importance of understanding the underlying mechanisms. Interestingly, plasma β-carotene levels appear to be a reliable biomarker of fruit and vegetable intake [38]. Despite the limitations of dietary recall methods, β-carotene intake assessed through these tools might still be a helpful indicator of fruit and vegetable consumption, which have been linked to better mental health in adolescents [17, 38]. This evidence suggests that β-carotene intake might be a marker for a more general healthy diet rich in fruits and vegetables, being associated with positive mental health outcomes.

In the current study, both higher β-carotene and vitamin C intakes were associated with fewer psychosomatic problems. While research specifically on nutrient intake is limited, existing research highlights the potential influence of diet patterns on these symptoms. For instance, the SEPAHAN cross-sectional studies found an association between healthier dietary patterns and lower psychosomatic problems in adults [39, 40]. The SEPAHAN study on the dietary inflammatory index showed that people with a more inflammatory diet were more likely to experience various psychosomatic symptoms including psychological, gastrointestinal, neuro-skeletal, and pharyngeal-respiratory somatic complaints, with vitamin C showing the strongest correlation among nutrients [39].

Unlike β-carotene’s link to all three mental health outcomes, the specific correlation between vitamin C and psychosomatic symptoms suggests that vitamin C might specifically target the physical manifestations of psychological distress rather than influencing overall mental health. However, this difference could be also due to limitations in study design or the need of a higher intake to see an overall effect. Studies investigating vitamin C supplementation at doses exceeding achievable dietary levels (500 mg–1 g per day) suggest a potential anxiolytic effect across different populations [41, 42]. However, there is a need for more studies involving a larger and more diverse group of participants to strengthen the evidence.

Our study did not find a significant association between vitamin E intake and any of the measured mental health outcomes. As already mentioned, a similar cross-sectional study reported no link between depression and anxiety and vitamin E intake in girls [29]. Additionally, a systematic review and meta-analysis on vitamin E supplementation have shown inconclusive results in improving anxiety [43]. As for β-carotene and vitamin C, no prior research has investigated the association between vitamin E intake, psychosomatic problems, and HRQoL. However, existing evidence on the use of vitamin E supplementation in combination with other nutrients, like omega-3s, suggests a potential influence of higher doses and synergistic effects of vitamin E on overall mental health [43, 44].

The lower intake of vitamin C for girls and β-carotene for both girls and boys, according to the recommendations, suggests a possible lower intake of fruits and vegetables, consistent with findings in Swedish adolescents [37, 45]. These findings are particularly relevant in the context of the observed gender differences in mental health, where girls reported significantly higher anxiety, psychosomatic symptoms, and lower HRQoL compared to boys. Research suggests that stress and negative emotions can influence dietary habits, potentially leading to both under- and overeating in response to psychological distress [46]. Some studies indicate that girls may be more prone to restrictive eating behaviours or lower overall energy intake during periods of high stress, which could contribute to inadequate consumption of antioxidant-rich foods [47]. These patterns may help explain the gender disparities in vitamin intake and their potential implications for mental well-being.

In addition to analysing individual vitamins, a summative vitamin intake score was examined to account for adherence to recommended vitamin intakes. The findings indicate that higher adherence to vitamin intake recommendations was significantly associated with fewer psychosomatic symptoms in the full sample and among boys. However, no significant associations were found between the vitamin intake scores and anxiety or HRQoL both in the whole sample and in the stratified analyses. As mentioned earlier, these findings may suggest that while certain vitamins could alleviate physical symptoms linked to stress, their impact on broader mental health, may require more specific nutritional interventions or higher doses than those measured in this study.

While a significant association between these antioxidants and mental health outcomes was observed, the study design does not allow for conclusions about the directionality of these associations, whether they reflect pre-existing vitamin deficiencies or other underlying factors. For example, vitamin C deficiency has been linked to psychosomatic symptoms such as anxiety, yet it remains unclear whether such symptoms precede or follow such deficiencies [48]. Moreover, individuals who consume more vitamin-rich foods may also engage in overall healthier eating habits, potentially confounding the observed associations.

Emerging research suggests a link between diet, mental health conditions and psychosomatic symptoms, involving oxidative stress and inflammation [39, 49]. Anxious individuals have shown elevated levels of free radicals and inflammatory markers such as C-reactive protein, tumour necrosis factor-α (TNF-α), interleukin-1β, and interleukin-6 (IL-6) compared to healthy controls [50, 51]. Dysregulation of nuclear factor kappa beta, a transcription factor involved in inflammation and immune responses, is linked to various psychological disorders and psychosomatic symptoms [52].

Given the role of oxidative stress and inflammation in mental health, antioxidants like vitamin C, E, and β-carotene may help mitigate these effects and positively influence mental health outcomes [28, 43, 53]. β-carotene combats free radicals, inhibits inflammatory signalling pathways and it has been shown to reduce the mRNA expression of IL-6 and TNF-α [28]. Similarly, vitamin C neutralizes free radicals, recycles other antioxidants and it might regulate mood-related neurotransmitters [54, 55]. Vitamin E protects cells from oxidation, boosts antioxidant enzyme activity, and lowers oxidative stress markers [43, 56].

This study has several strengths and limitations. A strength is that it involved a large sample, with an equal representation of boys and girls, and schools from smaller and larger municipalities and from urban and rural areas. In addition, the study employed validated methods for both dietary and mental health assessments. However, these assessments depend on self-reporting, which can be prone to errors due to recall bias or social desirability bias. Moreover, the 2-day food record window could have led to misclassifications. However, their impact on the results should be minimal given the large sample and a longer recall period might lead to participant fatigue and drop-outs. Additionally, antioxidants plasma levels were not measured, limiting the assessment of pre-existing deficiencies or absorption variations that might have influenced the associations. In addition, supplement use was not assessed. However, national data suggests that the use among this age group is very low (around 11%) [45]. Another limitation is that participants self-reported their gender by selecting between “girl,” “boy,” or “other.” While this approach is appropriate for the context, gender identity is more diverse than the provided options. A more inclusive approach, such as an open-ended question, may have been preferable. Residual confounding may also be present, as not all potential confounders were accounted for. Finally, this study design precludes establishing causality and cannot rule out reversed associations. Furthermore, generalizability might be limited to Nordic countries, because of potential similarities in terms of cultural dietary habits and supplement use patterns. Future studies with different populations and blood tests alongside self-reports could strengthen these aspects.

In conclusion, this cross-sectional study suggested a link between higher β-carotene intake, lower anxiety, fewer psychosomatic symptoms and better HRQoL. Higher vitamin C intake also appeared to be beneficial, especially for psychosomatic symptoms. While gender interactions were mostly non-significant, the findings highlight that overall antioxidant vitamin intake may play a role in psychosomatic symptoms, particularly among boys. Future research should replicate these results in diverse populations, incorporate biomarkers, and employ experimental designs to explore causal relationships. If confirmed, a link between diet and mental health could inform interventions promoting increased antioxidant intake from the diet among adolescents, potentially leading to improved mental well-being and a reduced burden of mental health problems in this population.

## Electronic supplementary material

Below is the link to the electronic supplementary material.

Supplementary Material 1

Supplementary Material 2

Supplementary Material 3

Supplementary Material 4

## Data Availability

The datasets are not available for download to protect the confidentiality of the participants. The data are held at The Swedish School of Sport and Health Sciences.
