# Supplementary material for: Associations between antioxidant vitamin intake and mental health in Swedish adolescents: a cross-sectional study
Source: Eur J Nutr. 2025 May 24;64(5):185. doi: 10.1007/s00394-025-03701-1 (PMC12103480; doi:10.1007/s00394-025-03701-1)
Supplement: Supplementary file 1 — Supplementary Material 1 [file 394_2025_3701_MOESM1_ESM.docx]

**Associations between antioxidant vitamin intake and mental health in Swedish adolescents: a cross-sectional study**

Martina Pensa, The Swedish School of Sport and Health Sciences, Stockholm, Sweden. [pensamartina18@gmail.com](mailto:pensamartina18@gmail.com)

Karin Kjellenberg, The Swedish School of Sport and Health Sciences, Department of Physical Activity and Health, Section for Health Science

Emerald Heiland, Uppsala University, Department of Surgical Sciences; Medical epidemiology; The Swedish School of Sport and Health Sciences, Department of Physical Activity and Health, Section for Health Science

Örjan Ekblom, The Swedish School of Sport and Health Sciences, Department of Physical Activity and Health, Section for Health Science

Gisela Nyberg, The Swedish School of Sport and Health Sciences, Department of Physical Activity and Health, Section for Health Science; Karolinska Instutitet, Department of Global Public Health, Karolinska Institutet

Björg Helgadóttir, The Swedish School of Sport and Health Sciences, Department of Physical Activity and Health, Section for Health Science


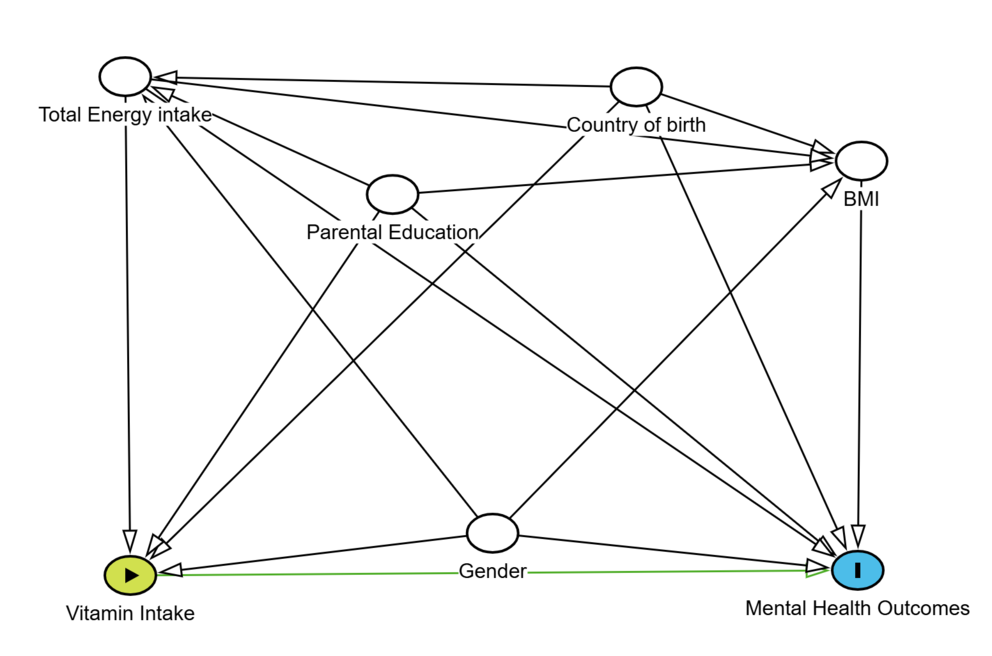


**Supplementary figure 1 Potential confounders and effect modifiers based on a Directed Acyclic Graph**

**Model code**

dag {

"Country of birth" [adjusted,pos="0.207,-1.464"]

"Mental Health Outcomes" [outcome,pos="1.126,1.557"]

"Parental Education" [adjusted,pos="-0.805,-0.792"]

"Total Energy intake" [adjusted,pos="-1.915,-1.528"]

"Vitamin Intake" [exposure,pos="-1.894,1.589"]

BMI [adjusted,pos="1.142,-1.000"]

Gender [adjusted,pos="-0.390,1.325"]

"Country of birth" -> "Mental Health Outcomes"

"Country of birth" -> "Total Energy intake"

"Country of birth" -> "Vitamin Intake"

"Country of birth" -> BMI

"Parental Education" -> "Mental Health Outcomes"

"Parental Education" -> "Total Energy intake"

"Parental Education" -> "Vitamin Intake"

"Parental Education" -> BMI

"Total Energy intake" -> "Mental Health Outcomes"

"Total Energy intake" -> "Vitamin Intake"

"Total Energy intake" -> BMI

"Vitamin Intake" -> "Mental Health Outcomes" [moderator="1"]

BMI -> "Mental Health Outcomes"

Gender -> "Mental Health Outcomes"

Gender -> "Total Energy intake"

Gender -> "Vitamin Intake" [moderator="1"]

Gender -> BMI

}
