# Supplementary material for: Associations between antioxidant vitamin intake and mental health in Swedish adolescents: a cross-sectional study
Source: Eur J Nutr. 2025 May 24;64(5):185. doi: 10.1007/s00394-025-03701-1 (PMC12103480; doi:10.1007/s00394-025-03701-1)
Supplement: Supplementary file 2 — Supplementary Material 2 [file 394_2025_3701_MOESM2_ESM.docx]

**Associations between antioxidant vitamin intake and mental health in Swedish adolescents: a cross-sectional study**

Martina Pensa, The Swedish School of Sport and Health Sciences, Stockholm, Sweden. [pensamartina18@gmail.com](mailto:pensamartina18@gmail.com)

Karin Kjellenberg, The Swedish School of Sport and Health Sciences, Department of Physical Activity and Health, Section for Health Science

Emerald Heiland, Uppsala University, Department of Surgical Sciences; Medical epidemiology; The Swedish School of Sport and Health Sciences, Department of Physical Activity and Health, Section for Health Science

Örjan Ekblom, The Swedish School of Sport and Health Sciences, Department of Physical Activity and Health, Section for Health Science

Gisela Nyberg, The Swedish School of Sport and Health Sciences, Department of Physical Activity and Health, Section for Health Science; Karolinska Instutitet, Department of Global Public Health, Karolinska Institutet

Björg Helgadóttir, The Swedish School of Sport and Health Sciences, Department of Physical Activity and Health, Section for Health Science


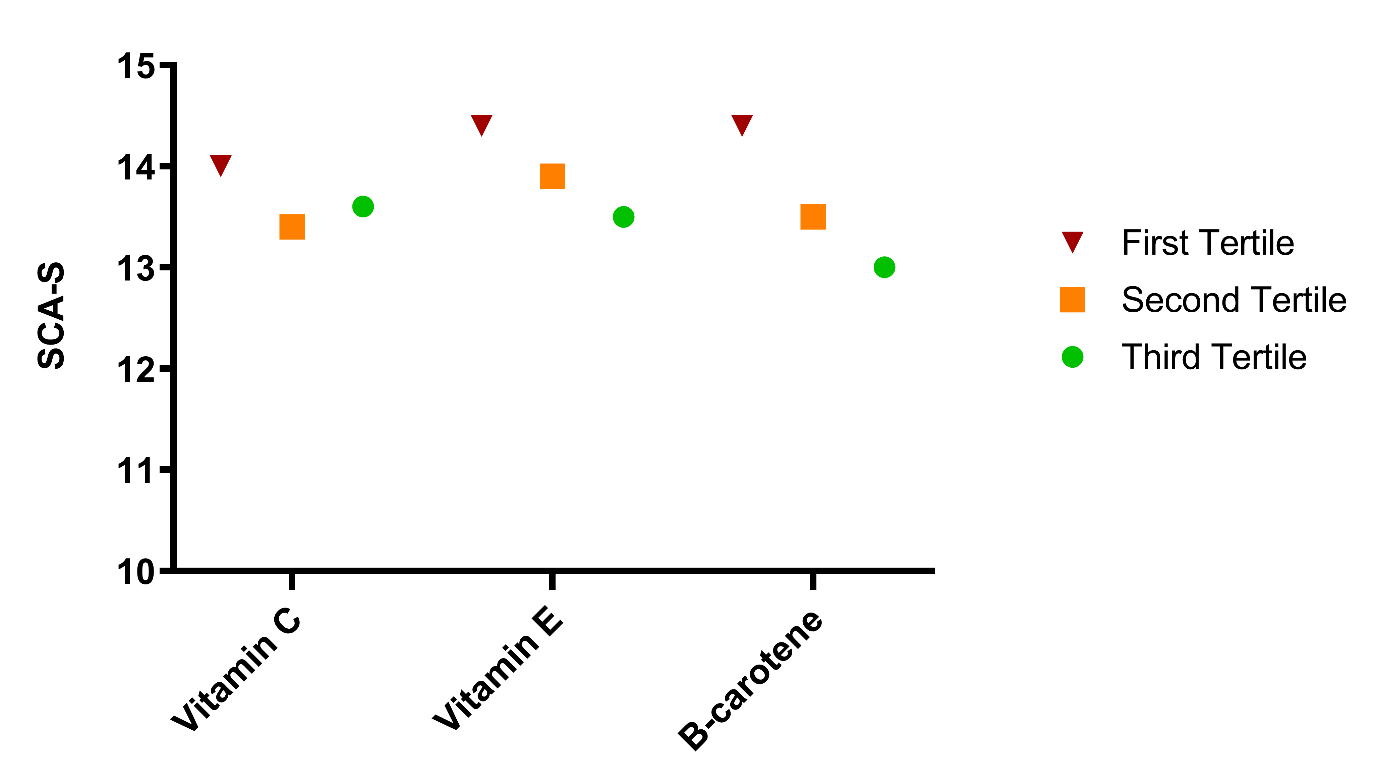


**Supplementary figure 1 Unadjusted mean SCAS-S (anxiety) scores between tertiles of vitamin C, E and β-carotene**


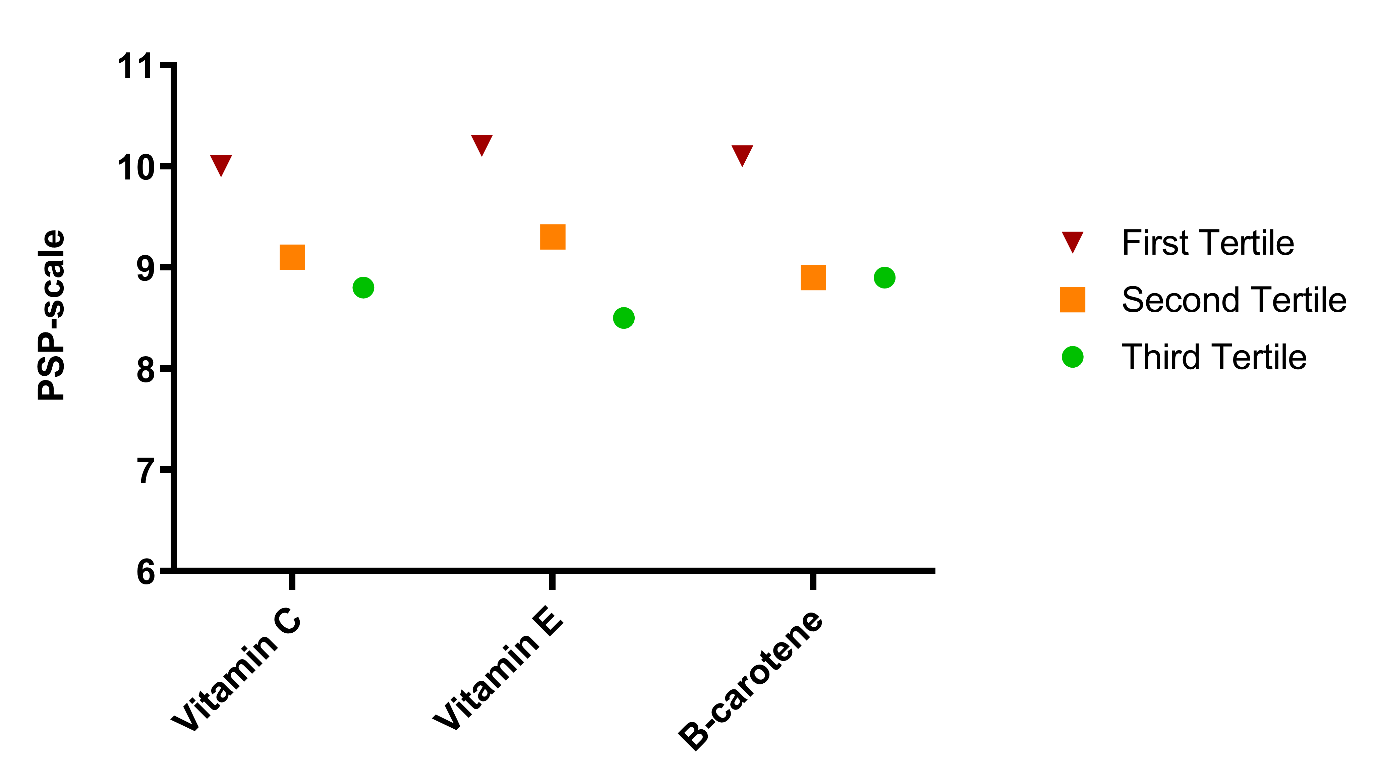


**Supplementary figure 2 Unadjusted mean PSP-scale (psychosomatic symptoms) scores between tertiles of vitamin C, E and β-carotene**


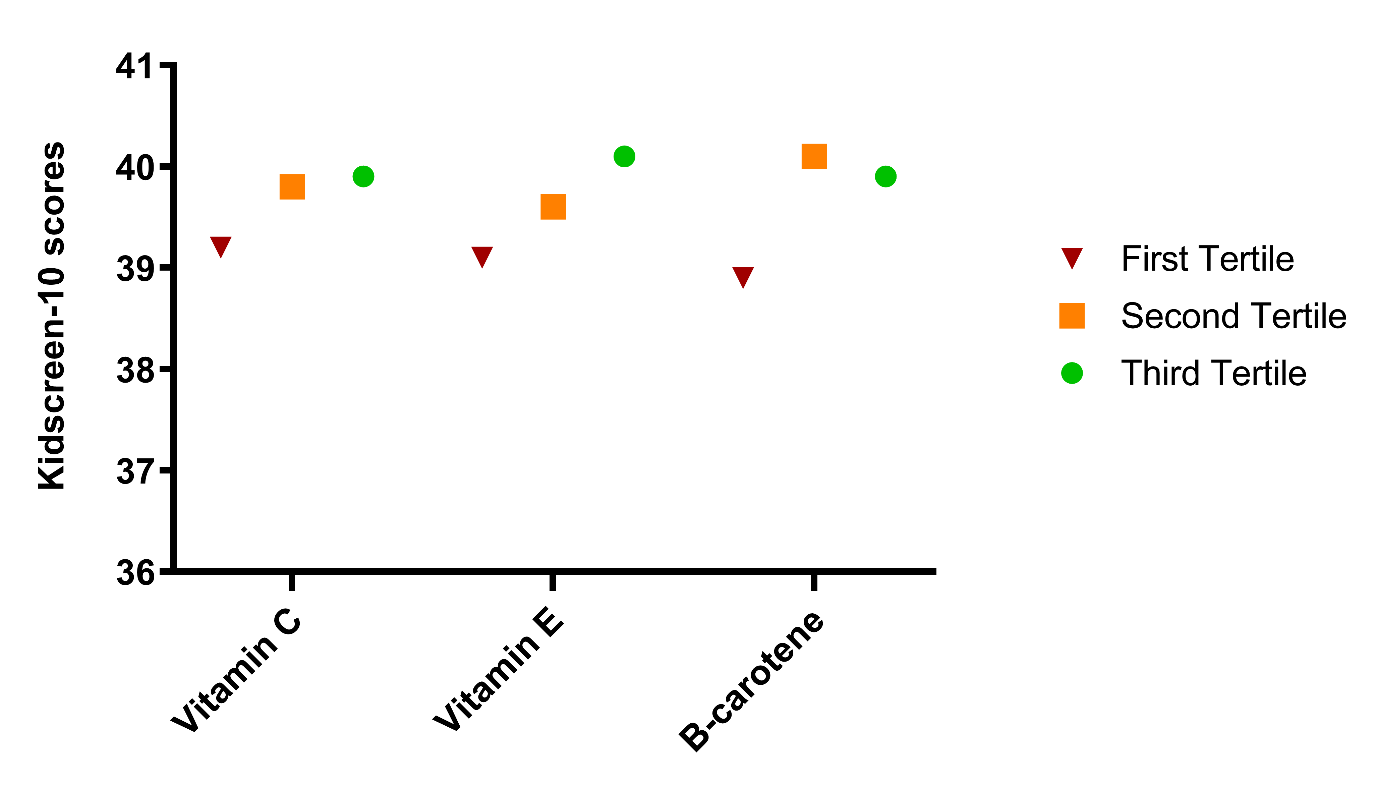


**Supplementary figure 3 Unadjusted mean Kidscreen-10 (HRQoL) scores between tertiles of vitamin C, E and β-carotene**
