# Supplementary material for: Associations between antioxidant vitamin intake and mental health in Swedish adolescents: a cross-sectional study
Source: Eur J Nutr. 2025 May 24;64(5):185. doi: 10.1007/s00394-025-03701-1 (PMC12103480; doi:10.1007/s00394-025-03701-1)
Supplement: Supplementary file 3 — Supplementary Material 3 [file 394_2025_3701_MOESM3_ESM.docx]

**Associations between antioxidant vitamin intake and mental health in Swedish adolescents: a cross-sectional study**

Martina Pensa, The Swedish School of Sport and Health Sciences, Stockholm, Sweden. [pensamartina18@gmail.com](mailto:pensamartina18@gmail.com)

Karin Kjellenberg, The Swedish School of Sport and Health Sciences, Department of Physical Activity and Health, Section for Health Science

Emerald Heiland, Uppsala University, Department of Surgical Sciences; Medical epidemiology; The Swedish School of Sport and Health Sciences, Department of Physical Activity and Health, Section for Health Science

Örjan Ekblom, The Swedish School of Sport and Health Sciences, Department of Physical Activity and Health, Section for Health Science

Gisela Nyberg, The Swedish School of Sport and Health Sciences, Department of Physical Activity and Health, Section for Health Science; Karolinska Instutitet, Department of Global Public Health, Karolinska Institutet

Björg Helgadóttir, The Swedish School of Sport and Health Sciences, Department of Physical Activity and Health, Section for Health Science

|  |  |  |  |  | 95% CI | |
| --- | --- | --- | --- | --- | --- | --- |
| Interaction term | Estimate | SE | t | p-value | Lower | Upper |
|  |  |  |  |  |  |  |
| Vitamin C |  |  |  |  |  |  |
| Tertile 1 x gender | Ref |  |  |  |  |  |
| Tertile 2 x gender | -0,11 | 0,18 | -0,58 | 0,561 | -0,46 | 0,25 |
| Tertile 3 x gender | -0,27 | 0,31 | -0,87 | 0,385 | -0,87 | 0,33 |
|  |  |  |  |  |  |  |
| Vitamin E |  |  |  |  |  |  |
| Tertile 1 x gender | Ref |  |  |  |  |  |
| Tertile 2 x gender | -0,27 | 0,23 | -1,20 | 0,231 | -0,71 | 0,17 |
| Tertile 3 x gender | -0,24 | 0,33 | -0,72 | 0,474 | -0,88 | 0,41 |
|  |  |  |  |  |  |  |
| β-carotene |  |  |  |  |  |  |
| Tertile 1 x gender | Ref |  |  |  |  |  |
| Tertile 2 x gender | -0,37 | 0,18 | -2,05 | **0,040*** | -0,73 | -0,02 |
| Tertile 3 x gender | -0,31 | 0,31 | -0,99 | 0,325 | -0,92 | 0,30 |

**Supplementary table 1 Interaction effects on the multiplicative scale between tertiles of vitamin intake and gender on anxiety symptoms (SCAS-S)**

SE, standard error, Ref, reference category

All results are adjusted for gender, BMIsds and total energy intake

* Significant results (p<0.05)

|  |  |  |  |  | 95% CI | |
| --- | --- | --- | --- | --- | --- | --- |
| Interaction term | Estimate | SE | t | p-value | Lower | Upper |
|  |  |  |  |  |  |  |
| Vitamin C |  |  |  |  |  |  |
| Tertile 1 x gender | Ref |  |  |  |  |  |
| Tertile 2 x gender | -0,31 | 0,13 | -2,40 | **0,017*** | -0,56 | -0,06 |
| Tertile 3 x gender | -0,41 | 0,22 | -1,90 | 0,058 | -0,83 | 0,01 |
|  |  |  |  |  |  |  |
| Vitamin E |  |  |  |  |  |  |
| Tertile 1 x gender | Ref |  |  |  |  |  |
| Tertile 2 x gender | -0,17 | 0,16 | -1,05 | 0,293 | -0,48 | 0,15 |
| Tertile 3 x gender | -0,25 | 0,23 | -1,05 | 0,295 | -0,70 | 0,21 |
|  |  |  |  |  |  |  |
| β-carotene |  |  |  |  |  |  |
| Tertile 1 x gender | Ref |  |  |  |  |  |
| Tertile 2 x gender | -0,23 | 0,13 | -1,77 | 0,076 | -0,48 | 0,02 |
| Tertile 3 x gender | -0,26 | 0,22 | -1,20 | 0,230 | -0,70 | 0,17 |

**Supplementary table 2 Interaction effects on the multiplicative scale between tertiles of vitamin intake and gender on psychosomatic symptoms (PSP-scale)**

SE, standard error, Ref, reference category

All results are adjusted for gender, BMIsds and total energy intake

* Significant results (p<0.05)

|  |  |  |  |  | 95% CI | |
| --- | --- | --- | --- | --- | --- | --- |
| Interaction term | Estimate | SE | t | p-value | Lower | Upper |
|  |  |  |  |  |  |  |
| Vitamin C |  |  |  |  |  |  |
| Tertile 1 x gender | Ref |  |  |  |  |  |
| Tertile 2 x gender | 0,14 | 0,13 | 1,05 | 0,295 | -0,12 | 0,39 |
| Tertile 3 x gender | 0,15 | 0,22 | 0,69 | 0,490 | -0,28 | 0,58 |
|  |  |  |  |  |  |  |
| Vitamin E |  |  |  |  |  |  |
| Tertile 1 x gender | Ref |  |  |  |  |  |
| Tertile 2 x gender | 0,07 | 0,16 | 0,42 | 0,673 | -0,25 | 0,38 |
| Tertile 3 x gender | 0,09 | 0,24 | 0,42 | 0,677 | -0,34 | 0,56 |
|  |  |  |  |  |  |  |
| β-carotene |  |  |  |  |  |  |
| Tertile 1 x gender | Ref |  |  |  |  |  |
| Tertile 2 x gender | 0,19 | 0,13 | 1,50 | 0,134 | -0,06 | 0,45 |
| Tertile 3 x gender | 0,25 | 0,22 | 1,11 | 0,269 | -0,19 | 0,68 |

**Supplementary table 3 Interaction effects on the multiplicative scale between tertiles of vitamin intake and gender on HRQoL (Kidscreen-10)**

SE, standard error, Ref, reference category

All results are adjusted for gender, BMIsds and total energy intake

* Significant results (p<0.05)

|  |  |  |  |  | 95% CI | |
| --- | --- | --- | --- | --- | --- | --- |
| Interaction term | Estimate | SE | t | p-value | Lower | Upper |
|  |  |  |  |  |  |  |
| Vitamin C |  |  |  |  |  |  |
| Tertile 1 x gender | Ref |  |  |  |  |  |
| Tertile 2 x gender | 0,51 | 0,95 | 0,53 | 0,594 | -1,36 | 2,38 |
| Tertile 3 x gender | -0,28 | 0,95 | -0,30 | 0,766 | -2,14 | 1,58 |
|  |  |  |  |  |  |  |
| Vitamin E |  |  |  |  |  |  |
| Tertile 1 x gender | Ref |  |  |  |  |  |
| Tertile 2 x gender | -1,04 | 0,95 | -1,09 | 0,274 | -2,91 | 0,83 |
| Tertile 3 x gender | 0,17 | 0,96 | 0,17 | 0,863 | -1,72 | 2,05 |
|  |  |  |  |  |  |  |
| β-carotene |  |  |  |  |  |  |
| Tertile 1 x gender | Ref |  |  |  |  |  |
| Tertile 2 x gender | 0,41 | 0,95 | 0,44 | 0,664 | -1,45 | 2,28 |
| Tertile 3 x gender | -0,21 | 0,95 | -0,22 | 0,824 | -2,08 | 1,66 |

**Supplementary table 4** **Interaction effects on the additive scale between tertiles of vitamin intake and gender on anxiety symptoms (SCAS-S)**

SE, standard error, Ref, reference category

All results are adjusted for BMIsds and total energy intake

* Significant results (p<0.05)

**Supplementary table** **5 Interaction effects on the additive scale between tertiles of vitamin intake and gender on psychosomatic symptoms (PSP-scale)**

|  |  |  |  |  | 95% CI | |
| --- | --- | --- | --- | --- | --- | --- |
| Interaction term | Estimate | SE | t | p-value | Lower | Upper |
|  |  |  |  |  |  |  |
| Vitamin C |  |  |  |  |  |  |
| Tertile 1 x gender | Ref |  |  |  |  |  |
| Tertile 2 x gender | -0,18 | 0,66 | -0,27 | 0,789 | -1,49 | 1,12 |
| Tertile 3 x gender | -0,01 | 0,66 | -0,02 | 0,982 | -1,32 | 1,29 |
|  |  |  |  |  |  |  |
| Vitamin E |  |  |  |  |  |  |
| Tertile 1 x gender | Ref |  |  |  |  |  |
| Tertile 2 x gender | 0,70 | 0,67 | 1,04 | 0,297 | -0,61 | 2,01 |
| Tertile 3 x gender | - 0,43 | 0,67 | -0,64 | 0,521 | -1,76 | 0,89 |
|  |  |  |  |  |  |  |
| β-carotene |  |  |  |  |  |  |
| Tertile 1 x gender | Ref |  |  |  |  |  |
| Tertile 2 x gender | 0,26 | 0,66 | 0,39 | 0,697 | -1,05 | 1,56 |
| Tertile 3 x gender | 0,55 | 0,67 | 0,82 | 0,410 | -0,76 | 1,86 |

SE, standard error

All results are adjusted for BMIsds and total energy intake

* Significant results (p<0.05)

|  |  |  |  |  | 95% CI | |
| --- | --- | --- | --- | --- | --- | --- |
| Interaction term | Estimate | SE | t | p-value | Lower | Upper |
|  |  |  |  |  |  |  |
| Vitamin C |  |  |  |  |  |  |
| Tertile 1 x gender | Ref |  |  |  |  |  |
| Tertile 2 x gender | -0,53 | 0,67 | -0,79 | 0,427 | -1,84 | 0,78 |
| Tertile 3 x gender | -0,09 | 0,67 | -0,13 | 0,898 | -1,40 | 1,23 |
|  |  |  |  |  |  |  |
| Vitamin E |  |  |  |  |  |  |
| Tertile 1 x gender | Ref |  |  |  |  |  |
| Tertile 2 x gender | 0,24 | 0,68 | 0,36 | 0,721 | -1,08 | 1,57 |
| Tertile 3 x gender | -0,03 | 0,68 | -0,04 | 0,967 | -1,36 | 1,30 |
|  |  |  |  |  |  |  |
| β-carotene |  |  |  |  |  |  |
| Tertile 1 x gender | Ref |  |  |  |  |  |
| Tertile 2 x gender | 0,45 | 0,67 | 0,67 | 0,504 | -0,87 | 1,76 |
| Tertile 3 x gender | -1,31 | 0,67 | -1,95 | 0,051 | -2,63 | 0,01 |

**Supplementary table 6 Interaction effects on the additive scale between tertiles of vitamin intake and gender on HRQoL (Kidscreen-10)**

SE, standard error

All results are adjusted for BMIsds and total energy intake

* Significant results (p<0.05)
