# Supplementary material for: Associations between antioxidant vitamin intake and mental health in Swedish adolescents: a cross-sectional study
Source: Eur J Nutr. 2025 May 24;64(5):185. doi: 10.1007/s00394-025-03701-1 (PMC12103480; doi:10.1007/s00394-025-03701-1)
Supplement: Supplementary file 4 — Supplementary Material 4 [file 394_2025_3701_MOESM4_ESM.docx]

**Associations between antioxidant vitamin intake and mental health in Swedish adolescents: a cross-sectional study**

Martina Pensa, The Swedish School of Sport and Health Sciences, Stockholm, Sweden. [pensamartina18@gmail.com](mailto:pensamartina18@gmail.com)

Karin Kjellenberg, The Swedish School of Sport and Health Sciences, Department of Physical Activity and Health, Section for Health Science

Emerald Heiland, Uppsala University, Department of Surgical Sciences; Medical epidemiology; The Swedish School of Sport and Health Sciences, Department of Physical Activity and Health, Section for Health Science

Örjan Ekblom, The Swedish School of Sport and Health Sciences, Department of Physical Activity and Health, Section for Health Science

Gisela Nyberg, The Swedish School of Sport and Health Sciences, Department of Physical Activity and Health, Section for Health Science; Karolinska Instutitet, Department of Global Public Health, Karolinska Institutet

Björg Helgadóttir, The Swedish School of Sport and Health Sciences, Department of Physical Activity and Health, Section for Health Science

**Table 1 Adjusted Linear Regression Analysis of Vitamin Intake Score and Mental Health Outcomes (Anxiety, Psychosomatic Symptoms, and HRQoL)**

| Score of vitamin intake | n | Anxiety |  | Psychosomatic  Symptoms |  | HRQoL |
| --- | --- | --- | --- | --- | --- | --- |
|  |  | β (95% CI) |  | β (95% CI) |  | β (95% CI) |
|  |  |  |  |  |  |  |
| All | 1133 |  |  |  |  |  |
| *Score 0 ^a^* | 325 | Ref |  | Ref |  | Ref |
| *Score 1 ^b^* | 451 | 0.30 (-0.87,1.46) |  | **-0.91 (-1.72,-0.09)*** |  | -0.04 (-0.87,0.79) |
| *Score 2 ^c^* | 286 | - 1. (-1.39,1.40) |  | **-1.19 (-2.16,-0.23)*** |  | -0.07 (-1.05,0.91) |
| *Score 3 ^d^* | 71 | - 1.17 (-1.27,1.48) |  | **-1.73 (-3.16,-0.29)*** |  | 1.30 (-0.16,2.76) |
|  |  |  |  |  |  |  |
| Girls | 580 |  |  |  |  |  |
| *Score 0* ^a^ | 167 | Ref |  | Ref |  | Ref |
| *Score 1 ^b^* | 239 | 1.07 (-0.70,2.85) |  | -0.66 (-1.87,0.56) |  | -0.17 (-1.32,0.99) |
| *Score 2 ^c^* | 144 | 1.15 (-0.96,3.27) |  | -0.61 (-2.06,0.84) |  | -0.05 (-1.86,0.89) |
| *Score 3 ^d^* | 30 | 0.19 (-3.10,3.48) |  | -0.95 (-3.20,1.30) |  | 1.19 (-0.94, 3.32) |
|  |  |  |  |  |  |  |
| Boys | 552 |  |  |  |  |  |
| *Score 0 ^a^* | 158 | Ref |  | Ref |  | Ref |
| *Score 1 ^b^* | 211 | -0.56 (-2.06,0.95) |  | **-1.15 (-2.24,-0.07)*** |  | 0.06 (-1.13,1.26) |
| *Score 2 ^c^* | 142 | -1.00 (-2.75,0.75) |  | **-1.76 (-3.03,-0.49)*** |  | 0.33 (-1.07,1.73) |
| *Score 3 ^d^* | 41 | -2.47 (-5.01,0.06) |  | **-2.48 (-4.29,-0.66)*** |  | 1.46 (-0.56,3.49) |

Ref, reference; CI, confidence interval; HRQoL, Health Related Quality of Life

^a^ Not meeting any recommended intakes for vitamin C, E or β-carotene

^b^ Meeting one of the recommended intakes for vitamin C, E or β-carotene

^c^ Meeting two of the recommended intakes for vitamin C, E or β-carotene

^d^ Meeting all the recommended intakes for vitamin C, E and β-carotene

* Significant results (p<0.05)
